# Systematic Review and Meta-Analysis of Labia Minora Anatomy in Premenopausal Women: Toward Better Labiaplasty Decisions

**DOI:** 10.3390/jcm15041641

**Published:** 2026-02-21

**Authors:** Isabel Ortega-Sánchez, María Orosia Lucha-López, Sofía Monti-Ballano

**Affiliations:** 1 Unidad de Investigación en Fisioterapia, Área de Antropología Social, Universidad de Zaragoza, Domingo Miral s/n, 50009 Zaragoza, Spain; iortega@unizar.es; 2 Unidad de Investigación en Fisioterapia, Spin off Centro Clínico OMT-E Fisioterapia SLP, Universidad de Zaragoza, Domingo Miral s/n, 50009 Zaragoza, Spain; smonti@unizar.es

**Keywords:** labiaplasty, labia minora, vulvar anatomy, anatomical variability, female genital cosmetic surgery, systematic review, meta-analysis, body image

## Abstract

**Background:** The labia minora are cutaneous folds richly innervated along their entire edge and are important for the protection of internal structures. Several studies have emphasized the wide interindividual variability in labia minora appearance. **Aim:** We aimed to conduct a systematic review and meta-analysis of research that has quantitatively described labia minora in healthy premenopausal adult women. **Methods:** Following Joanna Briggs Institute (JBI) and Preferred Reporting Items for Systematic Reviews and Meta-Analyses 2020 (PRISMA 2020) guidelines, we searched PubMed, Scopus, and Web of Science without date or language restrictions using the following terms and Boolean operators: (“labia minora” OR “vulva”) AND (anatom*) AND (measure* OR morphometr*). The search was last conducted on 27 October 2025. Observational studies reporting clinical measurements of labia minora length and/or width in women undergoing routine gynecological care were included. **Results:** The total number of records identified was 567. Seven cross-sectional studies comprising 991 women met the inclusion criteria. All studies collected measurements in hospital or outpatient clinical settings in Europe, Asia and the Middle East. All the measurements were conducted in the lithotomy position using standardized instruments. The range of labia minora length across studies varied from 36.5 mm to 60.6 mm. The labia minora width ranged from 14.7 mm to 21.8 mm. All studies described marked interindividual variability, with wide, overlapping ranges between samples. An important limitation concerns the incomplete assessment of measurement reliability in two of the included studies. A total of three studies, encompassing 307 women, provided the necessary data to permit meta-analysis. The pooled mean labia minora length was 53.23 mm (95% CI: 43.50–62.96), while the pooled mean labia minora width was 18.28 mm (95% CI: 14.93–21.64). The analysis of between-study variability revealed statistically significant heterogeneity (Cochran’s Q test, *p* < 0.001) and considerable heterogeneity (I^2^ > 75%) for both outcomes, i.e., labia minora length and width. **Conclusions:** The findings demonstrate substantial variability in labia minora dimensions. The integration of these data into medical education and clinical guidelines may be useful to reduce unnecessary interventions. Register: PROSPERO (registration number: CRD420251174590).

## 1. Introduction

The labia minora are cutaneous folds richly innervated along their entire edge and are important for sexual arousal [1]. Several studies have emphasized the wide interindividual variability in genital appearance [2]. Healthy and completely normal labia minora may be almost inconspicuous or protrude beyond the labia majora, may be asymmetric, or may be duplicated on one or both sides. Their skin may be smooth or slightly rugose and pigmented, particularly along the edges, and they exhibit marked sensitivity because they contain a considerable number of free nerve endings and sensory receptors [2]. They may range from 20 to 100 mm in length without causing any personal or cosmetic difficulty [3].

Despite this morphological diversity, demand for female genital cosmetic surgery (FGCS) has increased over the past two decades, and within this field, labiaplasty has become the most frequently performed procedure. According to the Global Survey on Aesthetic/Cosmetic Procedures (ISAPS), labiaplasty procedures increased by 112% between 2014 and 2024; this rise may in fact be even greater, given that most of these procedures are performed in the private healthcare sector, which is often less regulated [4,5]. This phenomenon occurs in a context in which there is no evidence of biological changes in the female population, such as a true increase in labia minora size or a higher prevalence of vulvar pathology, that would justify such growth [6,7].

The surgical practice of labiaplasty is justified on the basis of disparate definitions and shifting thresholds of what has been termed “labia minora hypertrophy”, which lacks a consensual diagnostic criterion and is sometimes defined by the linear dimensions of the labia minora in centimeters [8,9], and at other times by their aesthetic appearance, specifically, whether they protrude beyond the labia majora [10]. Some studies apply labial hypertrophy classification systems to recommend different labiaplasty techniques, such as deepithelialization when labia minora size is less than 20 mm (Type I) or between 20 and 40 mm (Type II), and other techniques for larger sizes [11]. In the absence of robust diagnostic criteria, the clinical decision to intervene surgically has shifted into the realm of contemporary aesthetic expectations, and many procedures are justified solely by the patient’s concern about her genital appearance [12].

As Lloyd et al. point out, there is an imbalance between the abundance of data on male genitalia and the scarcity of information on vulvar anatomy [3]. Classical medical literature and teaching materials offer a very limited representation of vulvar morphological diversity. Most textbooks and scientific articles depict the vulva with minimal variation and, although some studies have been published on clitoral size, vaginal length, or the internal structure of the clitoris in post-mortem dissections, there are few studies that systematically describe the dimensions of the labia minora [3]. This lack of objective data may contribute to the pathologization of normal anatomical variants and to surgical decision-making based on subjective judgments. Clinicians may not be sufficiently familiar with the full range of vulvar anatomical variation [13] and, in the absence of solid diagnostic criteria, may be influenced by emerging cultural and aesthetic norms when recommending or discouraging labiaplasty [14,15,16].

Given the lack of consensus on what constitutes a “normal” labia minora size and the need to provide rigorous information to counter unrealistic expectations, the aim of the present study is to conduct a systematic review and meta-analysis of research that has quantitatively described labia minora dimensions in healthy premenopausal adult women without vulvar pathology. By synthesizing these data, we aim to document the actual anatomical variability in this population, providing an empirical basis that may contribute to responsible clinical practice, improved medical education, and psychological and informational counseling that is more closely aligned with bodily diversity.

## 2. Materials and Methods

The present systematic review was conducted in accordance with the methodological guidelines established by the Joanna Briggs Institute, which provides comprehensive frameworks to support authors in undertaking systematic reviews [17].

The review process also adhered to the Preferred Reporting Items for Systematic Reviews and Meta-Analyses (PRISMA) 2020 statement, ensuring methodological rigor, transparency, and reproducibility in reporting [18]. The full PRISMA checklist is included as Supplementary Material (Table S1).

This systematic review was registered in the International Prospective Register of Systematic Reviews (PROSPERO) on 26 October 2025 (registration number: CRD420251174590). The registration details are available at https://www.crd.york.ac.uk/PROSPERO/view/CRD420251174590 (accessed on 23 October 2025).

ChatGPT (Free version) (OpenAI) (accessed on 24 November 2025) was used to assist in editing and improving the clarity of the English language throughout the manuscript.

### 2.1. Inclusion Criteria

Studies were eligible for inclusion if they fulfilled the predefined components of the Population, Exposure, and Outcome (PEO) framework, as recommended for systematic reviews of observational research:

- Population (P): Premenopausal adult women, irrespective of ethnicity or geographical location;
- Exposure (E): Routine vulvar examinations conducted in the absence of any pathological findings or vulvar disease;
- Outcome (O): Quantitative anatomical measurements of the labia minora, assessed through direct clinical examination.

Eligible study designs included descriptive cross-sectional studies and analytical observational studies, such as prospective cohort studies, retrospective cohort studies, and case–control studies. Studies were included regardless of publication year or language, provided that sufficient methodological and outcome data could be extracted.

### 2.2. Exclusion Criteria

Studies were excluded if they did not meet the predefined PEO components or lacked extractable data relevant to the outcomes of interest. The following types of publications were also excluded: single case reports, case series, narrative or systematic reviews, commentaries, expert consensus documents, editorials, short communications, and letters to the editor.

Articles not available in full text, as well as those without clearly defined participant characteristics or outcome measures, were also excluded.

### 2.3. Information Sources and Search Strategy

The following electronic databases were systematically searched to identify relevant studies: PubMed, Scopus, and Web of Science. The search strategies were designed and adapted to the specific syntax and indexing terms of each database to ensure optimal retrieval. Boolean operators (“AND”, “OR”), truncation symbols (*), and field tags were applied as appropriate. Controlled vocabulary terms (e.g., MeSH in PubMed) were combined with free-text keywords to enhance sensitivity and specificity.

The final search strategies were as follows:

- PubMed: (“labia minora” OR “vulva”) AND (anatom*) AND (measure* OR morphometr*);
- Scopus: TITLE-ABS-KEY((“labia minora” OR “vulva”) AND (anatom*) AND (measure* OR morphometr*));
- Web of Science: ((((TS = “labia minora”) OR (TS = “vulva”)) AND (TS = anatom*)) AND ((TS = measure*) OR (TS = morphometr*))).

No restrictions were applied regarding publication date, study design, or language to minimize selection bias. The search was last conducted on 27 October 2025.

In addition to database searches, reference lists of all eligible studies were manually screened to identify any additional studies not captured through electronic databases.

### 2.4. Selection Process

All retrieved references were imported into Mendeley Reference Manager for organization and deduplication prior to screening.

The study selection process was carried out in two sequential phases.

In the first phase, the titles and abstracts of all identified records were independently screened by two reviewers (M.O.L.-L. and S.M.-B.) to identify potentially eligible studies based on the predefined inclusion and exclusion criteria. In the second phase, the full texts of the selected articles were examined in detail to determine their final eligibility.

Any disagreements or uncertainties arising during the screening or full-text assessment were resolved through discussion, with a third reviewer (I.O.-S.) consulted when necessary to achieve consensus. The entire process was documented and will be presented in a PRISMA 2020 [18] flow diagram to ensure transparency and reproducibility.

### 2.5. Data Collection Process

Data from all studies meeting the eligibility criteria were extracted independently by two reviewers (M.O.L.-L. and S.M.-B.). Discrepancies between reviewers were resolved by discussion, and a third reviewer (I.O.-S.) was consulted when consensus could not be reached.

### 2.6. Data Items

The extracted information included the following:

- Study characteristics: author(s), year of publication, country, study design, sample size, and setting.
- Population characteristics: age range, ethnicity, and other relevant demographic details.
- Exposure details: type and methodology of routine vulvar examination performed.
- Outcome measures: quantitative anatomical measurements (length and width) of the labia minora and reported statistical data. Different measurement techniques may be used across studies (e.g., different instruments, use of anesthesia versus awake examinations), which could influence the reported dimensions. While this may increase heterogeneity, studies were included regardless of measurement method in order to maximize the number of studies and provide a more comprehensive overview of the available evidence. Different measurement approaches are acceptable provided that they are standardized, clearly described, and employ appropriate instruments, ensuring that the data remain interpretable.
- Additional information: study limitations and key findings.

All extracted data were compiled into a centralized database (Microsoft Excel), which facilitated consistency checks, verification, and preparation for subsequent qualitative and quantitative synthesis.

### 2.7. Study Risk of Bias Assessment

The risk of bias was evaluated using the critical appraisal tools developed by the Joanna Briggs Institute (https://jbi.global/critical-appraisal-tools; last accessed on 29 October 2025). Based on a thorough assessment of the study designs, the critical appraisal instrument for analytical cross-sectional [19] was selected as the most appropriate for the included studies. This instrument comprises eight items that systematically examine essential methodological elements indicative of a high-quality cross-sectional study:

1. Were the criteria for inclusion in the sample clearly defined?
2. Were the study subjects and the setting described in detail?
3. Was the exposure measured in a valid and reliable way?
4. Were objective, standard criteria used for measurement of the condition?
5. Were confounding factors identified?
6. Were strategies to deal with confounding factors stated?
7. Were the outcomes measured in a valid and reliable way?
8. Was appropriate statistical analysis used?

The possible responses are: Yes, No, Unclear, Not applicable.

### 2.8. Data Synthesis and Statistical Analysis

Data were organized using Microsoft Excel 16.78.3. The results of each individual study, as well as the synthesized quantitative findings, were presented in consolidated tables.

A meta-analysis of single means was performed to obtain pooled estimates of labia minora length and width. Measurements of the length and width of the labia minora were included when obtained using a valid and reliable methodology, specifically with a measuring tape or calipers, recorded in centimeters or millimeters. Measurements recorded in centimeters were converted to millimeters. When measurements of the labia minora were reported separately for the left and right sides, their average was computed to derive a single representative value. Only studies reporting the mean, standard deviation, and sample size were included in the quantitative synthesis.

Statistical analyses were conducted using R (version 4.5.2) with the metafor package. Pooled means and corresponding 95% confidence intervals were calculated for each outcome. Random-effects models were applied to account for between-study variability. Statistical heterogeneity was assessed using Cochran’s Q test, the I^2^ statistic, and τ^2^.

The *p*-value of Cochran’s Q test was used to assess whether the observed heterogeneity exceeded that expected by chance alone. Cochran’s Q test evaluates the null hypothesis (H_0_) that all studies share a common true mean, against the alternative hypothesis (H_1_) that at least one study-specific true mean differs. The null hypothesis was rejected when the *p*-value was ≤0.05.

The I^2^ statistic was used to quantify the proportion of total variability attributable to between-study heterogeneity. Conventional reference thresholds were applied for descriptive purposes (0–25%: low heterogeneity; 25–50%: moderate heterogeneity; 50–75%: substantial heterogeneity; >75%: considerable heterogeneity).

Between-study variance (τ^2^) was estimated using restricted maximum likelihood (REML), which provides less biased variance estimates than conventional methods, particularly when the number of studies is small. τ^2^ represents the variability in true mean estimates across studies. The square root of τ^2^ (τ) was used to quantify the typical deviation of study-specific true means from the pooled mean, allowing a clinically meaningful interpretation of between-study variability.

Forest plots were created to visually display individual study means with their corresponding 95% confidence intervals, together with the pooled mean estimated using a random-effects model (REML). Separate forest plots were generated for labia minora length and labia minora width.

## 3. Results

### 3.1. Study Selection

**Figure 1:**
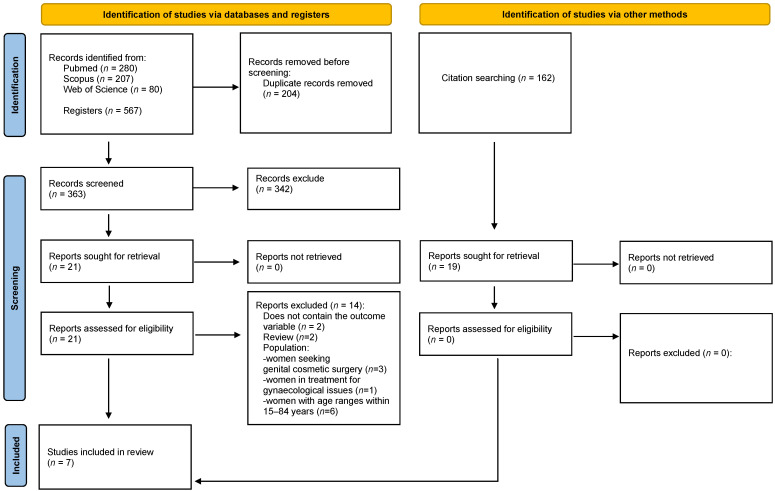
PRISMA 2020 [18] flow diagram.

Each database was last searched on 6 November 2025. The number of records identified was as follows: PubMed (*n* = 280), Scopus (*n* = 207), and Web of Science (*n* = 80); these databases yielded a total of 567 records. Following the removal of duplicates and screening of titles and abstracts, 21 records were selected for full-text retrieval. All 21 records were successfully retrieved and assessed for eligibility. Upon full-text evaluation, seven studies met the predefined eligibility criteria and were included in the Section 3. No additional studies were identified through reference list screening of the eligible articles (Figure 1).

### 3.2. Study Characteristics and Results of Individual Studies

The seven articles ultimately included in the review were all analytical cross-sectional studies, and their results are summarized in Table 1.

Lloyd et al. and Basaran et al. each examined samples of 50 premenopausal women [3,20], while Krissi et al. included 32 participants [21], Lykkebo et al. evaluated 244 women [22], Kaya et al. studied 208 women [23], Mangla et al. included 207 women [24], and Kurtoglu et al. examined 200 participants [25]. All studies collected measurements in hospital or outpatient clinical settings in Europe, Asia and the Middle East. Overall, these findings demonstrate substantial variability in labia minora dimensions.

All measurements were performed in the lithotomy position using standardized instruments. A detailed description of the vulvar examination methodology is provided in Table 2.

### 3.3. Risk of Bias in Studies

Table 3 presents the results of the risk of bias analysis for all included studies.

The eight criteria of the critical appraisal tool for analytical cross-sectional studies from the Joanna Briggs Institute (https://jbi.global/critical-appraisal-tools; last accessed on 29 October 2025) were applied to the selected studies.

The only question that indicated a potential risk of bias was ‘Were the outcomes measured in a valid and reliable way?’. In the studies by Basaran et al. and Krissi et al. [20,21], data on reliability or validity based on previous literature were not provided, nor was it clarified in the text if any measures were taken to ensure the reliability of the measurements. Therefore, some potential bias regarding the reliability of the measurements may exist in these two studies.

All other criteria were adequately fulfilled by all studies, allowing us to confidently assess the methodological quality of the selected studies.

### 3.4. Results of Syntheses

Table 4 presents a comprehensive synthesis of the quantitative extracted data pertaining to the outcome variables, namely the length and width of the labia minora.

A total of three studies [3,20,24], comprising 307 women, were included in the quantitative analysis. The remaining studies did not provide data on standard deviations and, consequently, were excluded from the meta-analysis.

Taking these considerations into account, the pooled mean labia minora length was 53.23 mm (SE: 4.97 mm; 95% CI: 43.50–62.96). Cochran’s Q test was statistically significant, indicating the presence of between-study heterogeneity beyond sampling error (Q = 79.27, *p* < 0.001). A total of 96.98% of the observed variability was attributable to between-study heterogeneity (I^2^ = 96.98%). The true study-level means differed from one another with a standard deviation of 8.43 mm, as reflected by the estimated between-study variance (τ^2^ = 70.98 mm^2^; τ = 8.43 mm).

**Figure 2:**
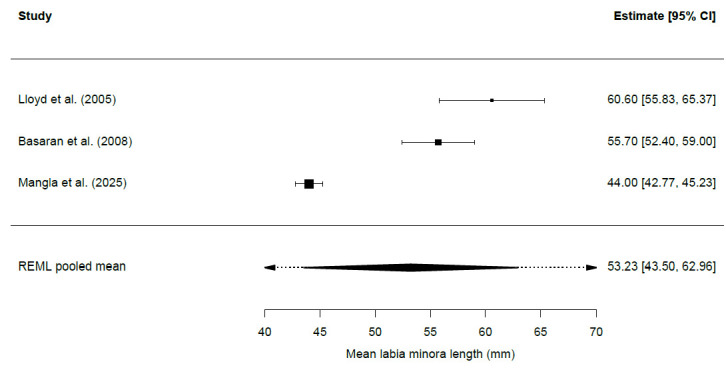
Forest plot showing study-level mean estimates and pooled mean for labia minora length (mm). Individual study means with 95% confidence intervals are displayed. The pooled estimate was calculated using a random-effects model with restricted maximum likelihood (REML) estimation [3,20,24].

In Figure 2, we can see the forest plot showing individual study mean estimates and the pooled mean for labia minora length (mm).

The pooled mean labia minora width was 18.28 mm (SE: 1.71 mm; 95% CI: 14.93–21.64). Cochran’s Q test was also statistically significant, indicating substantial between-study heterogeneity beyond sampling error (Q = 22.99, *p* < 0.001). Overall, 94.64% of the total observed variability was attributable to heterogeneity between studies (I^2^ = 94.64%). The true study-specific means varied with a standard deviation of 2.84 mm, corresponding to a between-study variance of τ^2^ = 8.07 mm^2^ (τ = 2.84 mm).

**Figure 3:**
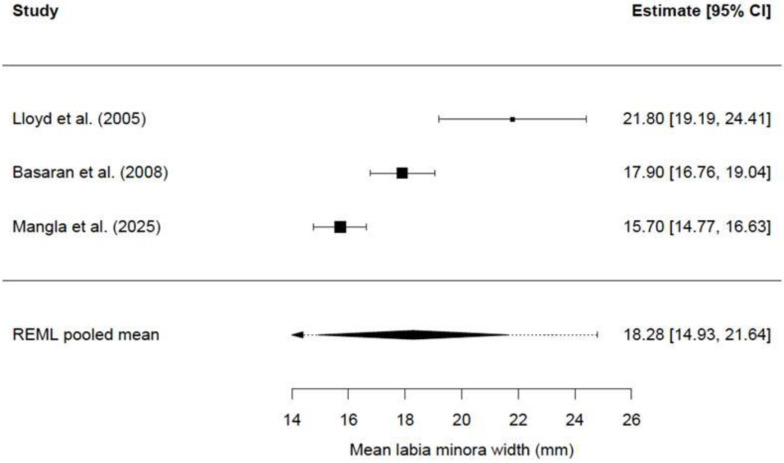
Forest plot showing study-level mean estimates and the pooled mean for labia minora width (mm). Individual study means with 95% confidence intervals are displayed. The pooled estimate was calculated using a random-effects model with restricted maximum likelihood (REML) estimation [3,20,24].

In Figure 3, we can see the forest plot showing individual study mean estimates and the pooled mean for labia minora width (mm).

## 4. Discussion

This systematic review and meta-analysis documents the wide anatomical variability in the labia minora in premenopausal adult women without vulvar pathology. These results, derived from seven observational studies that included 991 women, and the meta-analysis of three studies including 307 women, confirm the interindividual variability highlighted by previous studies. The wide dispersion of values observed in the series by Lloyd and Başaran is repeated in more recent and larger studies, such as those by Lykkebo and Mangla.

These findings reinforce the notion that there is no single or “normal” anatomical measurement for the labia minora, but rather a wide spectrum of morphologies; therefore, attempts to define “hypertrophy” solely on the basis of linear measurements in centimeters are not consistently supported by available empirical evidence, and labial dimensions alone cannot be interpreted as indicators of malformation in the absence of pathology. Nevertheless, numerous studies set a dimension of 40–50 mm (measured from the base of insertion to the free edge) to define hypertrophy and recommend labiaplasty [8,9,10,11], and some critical works denounce that the sizes of labia minora subjected to labiaplasty are highly variable. For example, Michala et al. found that the mean width of the labia minora operated on was 36 mm (range 20–55 mm), and in cases of asymmetry, the difference between one side and the other ranged from 6 to 35 mm, with a mean of 20 mm [26].

The results of our study show that the pooled mean labia minora width was 18.28 mm, with a 95% confidence interval ranging from 14.93 to 21.64 mm. Some studies apply labial hypertrophy classification systems to guide surgical decision-making, recommending different labiaplasty techniques when labia minora size is less than 20 mm (Type I) or between 20 and 40 mm (Type II), and other techniques for larger sizes [11]. In this context, a proportion of labiaplasty procedures may be performed in women whose labia minora width overlaps with ranges of anatomical variability described in populations without vulvar pathology.

The analysis of between-study variability revealed statistically significant heterogeneity (Cochran’s Q test, *p* < 0.001) and considerable heterogeneity (I^2^ > 75%) for both outcomes, labia minora length and width. Given the very limited number of included studies and the extremely high heterogeneity, the pooled estimates should be interpreted with caution and considered exploratory rather than definitive. Accordingly, the narrative synthesis should be prioritized to contextualize individual study findings and to provide a more nuanced interpretation of the available evidence. Part of the observed heterogeneity can be interpreted as reflecting true biological variability in labia minora dimensions across populations. However, some of this variability may also be influenced by differences in sociodemographic characteristics of the source populations (such as ethnicity and parity) and by variations in measurement techniques (e.g., different instruments, use of anesthesia versus awake examinations). Despite these factors, the resulting heterogeneous pattern enhances the generalizability of the findings to diverse populations of premenopausal women, which aligns with the primary objective of this review.

In this meta-analysis, τ indicated that true study-level means typically differed from the pooled estimate by approximately 8.43 mm for labia minora length and 2.84 mm for labia minora width. Given that the quantitative findings of this review are descriptive and derived from observational studies with high interindividual variability, the following interpretations should be understood as contextualized implications rather than direct causal inferences. The considerable variability in labia minora measurements across the population, along with the lack of universally established anatomical thresholds, suggests that dimensional data alone may not fully capture the clinical or experiential nuances of labiaplasty. In this context, an integrative framework incorporating patient-reported experiences, perceptions and contextual factors may be informative when interpreting anatomical data, as described in the qualitative literature. Within this broader perspective, clinical narratives surrounding labiaplasty provide valuable insights into the motivations and expectations of patients.

Regarding possible functional discomfort related to size, the available evidence across the seven included studies indicates that, at the population level, the anatomical dimensions of the labia minora are not consistently associated with sexual function or clinical dysfunction. Variations in size, shape, or asymmetry are generally described as anatomically normal and functionally neutral. Nevertheless, individual women may still report symptoms such as irritation, chafing, or localized discomfort. These symptoms do not appear to follow a linear or size-dependent pattern and are not consistently predicted by anatomical measurements alone.

None of the included studies associates morphological variability with dysfunction or genital pathology. The study by Lloyd et al. [3] notes that many women with protruding or asymmetric labia minora were completely asymptomatic, demonstrating that protrusion is not linked to physical discomfort. Basaran et al. [20] report no functional alterations derived from anatomical size in premenopausal women, while Mangla et al. [24] document that participants with greater protrusion or asymmetry experienced neither discomfort nor dysfunction, and the authors conclude that functional complaints—such as chafing or discomfort—do not have a linear relationship with anatomical dimensions.

In those studies that evaluated the relationship between size and sexual function, no statistically significant correlation between anatomical measurements and sexual function scores was observed, suggesting that aesthetic perception and body self-image may influence sexual well-being more than objective anatomical measures [21,23]. Thus, even when healthy women report discomfort that is mainly psychological or culturally mediated, available evidence indicates that labia minora size alone is not consistently associated with functional impairment and, in isolation, does not constitute a reliable anatomical criterion for defining “hypertrophy” or for establishing surgical indication. Several studies show that a negative self-perception of genital appearance may be associated with lower sexual satisfaction, although without a demonstrable anatomical basis [22,23,25].

It is notable that labiaplasty is often promoted as a ‘genital rejuvenation’ procedure, when the size of the labia minora is smaller in postmenopausal women than in premenopausal women [20]. This suggests that the rejuvenation discourse is less associated with biological age than with an aesthetic ideal of prepubertal appearance.

Some studies support the benefits of labiaplasty and consider it a safe procedure [27,28]; others have concluded that, although labiaplasty is generally safe, severe complications have been described, in some cases requiring additional surgical interventions [29]. Prospective studies that collected complications reported directly by patients instead of by their physicians documented higher complication rates [30]. It cannot be overlooked that this intervention affects tissue that significantly contributes to sensory sexual arousal, requiring special clinical caution [31].

Taken together, these findings reinforce the idea that realistic, visually inclusive, and culturally sensitive genital education is essential to support ethical clinical practice, promote healthy body image, and depathologize female anatomical variability.

The present study is subject to several limitations. Regarding the evidence synthesized in this review, an important limitation concerns the incomplete assessment of measurement reliability in two of the included studies. In addition, some aspects of measurement standardization varied across studies, including the use of anesthesia and measurement instruments. Another limitation relates to the heterogeneity of the study populations, which were drawn from different geographical settings, encompassed diverse ethnic backgrounds, and differed in obstetric characteristics. Although these factors may plausibly influence anatomical dimensions, formal adjustment through meta-regression was not undertaken because the limited number of available studies would not allow reliable estimation of covariate effects. Future studies with standardized methodologies and larger sample sizes are needed before robust reference values can be established. Until such data are available, the present findings should be interpreted as preliminary and descriptive.

The small number of included studies also limits the feasibility of formal assessments of publication bias; therefore, publication bias cannot be definitively ruled out.

Despite these limitations, we believe that the findings of this meta-analysis are informative, as they provide a quantitative framework supporting the broad range of normal variability in labia minora morphology among healthy premenopausal adult women without vulvar pathology. This framework is likely to be broadly generalizable across diverse populations.

In light of the wide anatomical variability identified in the present meta-analysis and the absence of consistent anatomical thresholds associated with functional outcomes, the following section explores the clinical and sociocultural factors that may help contextualize the demand for labiaplasty.

### Clinical and Sociocultural Implications

The available literature indicates that, rather than functional discomfort, genital perception—shaped by contemporary aesthetic ideals, visual media, and pornography—may play a central role in shaping the demand for labiaplasty. The ideal of a ‘flattened genital surface,’ characterized by the absence of labia minora protrusion, has become a normative aesthetic model in social networks and clinical–aesthetic environments [3]. As a result, many adolescents and adult women express concern about the appearance of their vulva and seek medical consultation to assess their anatomical normality [26,32]. Clinical studies of women requesting labiaplasty have shown that they typically present morphological variations in the labia minora that fall within normal anatomical parameters [6,16].

Psychological distress related to genital appearance is a frequent motivation for consultation [30,33]. This distress may manifest as “pudendal disgust” or “pudendal self-loathing,” a psychosexual response associated with shame and aversion toward one’s own genitalia, often arising from comparison with unattainable aesthetic ideals [34]. Body dysmorphia may also be present, and surgical intervention may reinforce, rather than resolve, these sociocultural and psychological dynamics by further narrowing the range of what is considered acceptable anatomy [5].

The reviewed articles mention the role of visual media in genital perception. Other works also relate exposure to schematic representations of the vulva and messages equating labial visibility with abnormality to negative genital body image. The representation of female genital anatomy in the media, erotic magazines, and pornography is characterized by a very narrow range of shapes and sizes [35,36].

Several studies have questioned the accuracy of the information and content disseminated on genital cosmetic surgery websites in countries such as the United States and the United Kingdom [37], as well as in Belgium, Canada, and Brazil [38]; Australia [39,40]; and Spain [41], showing a discourse that pathologizes anatomical variations in the vulva through the use of ‘before and after’ photographs that emphasize a purportedly ‘natural’ aesthetic outcome after surgery [37]. Moreover, increasing exposure to representations of surgically modified vulvas may shape standards of normality and contributes to body dissatisfaction among healthy women seeking labial reduction [42].

In this context, the findings of this review suggest that strengthening anatomical, sexual, and medical education on vulvar diversity, together with appropriate psychological support, may represent relevant alternatives to an immediate surgical response. Persistent ignorance of female anatomical variability among both patients and healthcare professionals may reinforce distorted notions of normality and unrealistic expectations [3,22]. Normalizing anatomical diversity and promoting inclusive representations may help alleviate body-related distress and support more informed and autonomous decision-making.

## 5. Conclusions

Overall, this review provides empirical evidence that may contribute to a broader understanding of the range of anatomical variation and may support clinical approaches that are attentive to female anatomical diversity. The term “hypertrophy” warrants careful consideration, as its use may inadvertently contribute to the medicalization of normal bodily variation. Methodological heterogeneity across studies, including differences in measurement techniques and study populations, may also contribute to the observed variability in reported anatomical dimensions.

Incorporating these findings into medical education, informed consent processes, and discussions surrounding clinical practice in genital cosmetic surgery may help to contextualize anatomical variation and support a more nuanced appreciation of female anatomy, while acknowledging the need for further high-quality research. The availability of anatomical measurements may have educational value, provided they are used for informational rather than normative purposes. Finally, decisions regarding surgical intervention should take into account symptoms, functional outcomes, and fully informed shared decision-making, rather than anatomical measurements alone.

## Figures and Tables

**Table 1:** Summary of individual included studies.

Study: Author(s), Year of Publication	Study Characteristics: Study Design, Sample Size, Setting, Country	Population Characteristics: Age (Years) and Other Relevant Demographic Details	Outcome Measures: Reported Statistical Data on the Quantitative Anatomical Measurements of the Labia Minora	Additional Information: Study Limitations and Key Findings
Lloyd et al. (2005) [3]	Observational cross-sectional study (*n* = 50); women undergoing routine procedures (hysteroscopy or diagnostic laparoscopy) in a hospital setting, United Kingdom.	Premenopausal women (mean 35.6 ± 8.7 years (18–50); predominantly White (*n* = 37), Asian (*n* = 5), Black (*n* = 6), Latin American (*n* = 1), mixed-race (*n* = 1); nulliparous (*n* = 29), parous (*n* = 18).	Labia minora length (mm): 60.6 ± 17.2, (20–100). Labia minora width (mm): 21.8 ± 9.4, (7–50).	There is considerable variation in genital dimensions among women.
Basaran et al. (2008) [20]	Observational cross-sectional study (*n* = 50); outpatient gynecology and menopause clinics in a hospital setting, Turkey.	Premenopausal women; Age 30.2 ± 4.2 (22–39) years; Gravida 2.1 ± 1.2 (0–5); Parity 1.7 ± 0.8 (0–4).	Labia minora length (mm): Left 55.8 ± 12.3 (35–75); Right 55.6 ± 11.6 (33–75); Mean 55.7 ± 11.9 (34–74). Labia minora width (mm): Left 18.1 ± 4.6 (12–33); Right 17.7 ± 4.8 (9–29); Mean 17.9 ± 4.1 (11–30).	In a carefully selected cohort, normative anatomical values were determined.
Krissi et al., (2016) [21]	Cross-sectional prospective cohort study (*n* = 32); women undergoing diagnostic hysteroscopy for an indication unrelated to the vulvar or vaginal morphology in a hospital setting, Israel.	Premenopausal women. Age: 33.38 ± 7.95 years (20–51); BMI: 23.93 ± 4.21 (16.6–33.9); Parity: 62.4% parous; Vaginal delivery 46.8% (*n* = 15); Cesarean 15.6% (*n* = 5).	Labia minora length (mm): Right 34.7 ± 14.2 (10–60); Left 38.2 ± 13.3 (20–60). Labia minora width (mm): Right 14.9 ± 10.3 (10–30); Left 14.5 ± 8.7 (10–40).	Despite limitations in sample size, age range and parity, this study provides valuable data on genital variability.
Lykkebo et al. (2017) [22]	Cross-sectional study (*n* = 244); outpatient obstetrics and gynaecology clinics in a hospital setting, Denmark. Women visiting the outpatient clinic for different reasons.	Premenopausal white women. Age: years (18–50); BMI: Mean 24, 95% CI: 23.4–24.5, (17.3–45.3); Parity: Mean 1.4, 95% CI: 1.2–1.6, (0–5).	Labia minora length (mm): Mean 42.8, 95% CI: 41.2–44.4, (5–82). Labia minora width (mm): Mean 15.5, 95% CI: 14.4–16.5, (1–40).	Women seeking labiaplasty may hold misperceptions regarding normal genital appearance and express concern about the labia minora. Access to accurate education and information on the diversity of normal genital morphology is essential.
Kaya et al. (2020) [23]	Cross-sectional study (*n* = 208); outpatient gynaecology clinic in a hospital setting, Turkey. Routine gynecologic examinations.	Premenopausal women; Age 35.2 ± 9.1 (18–52) years; BMI (kg/m^2^): 25.1 ± 4.6 (16.3–41.5). Parity: Nulliparous 17.3% (*n* = 36); Multiparous 82.7% (*n* = 172. Multiparous delivery mode: Vaginal 58.4% (*n* = 101); Cesarean 32% (*n* = 55); Both 9.3% (*n* = 16).	Labia minora length (mm): Right: 36.0 ± 11.7 (15–78); Left: 37.9 ± 12.6 (19–75). Labia minora width (mm): Right: 21.2 ± 8.6 (6–79); Left: 22.0 ± 9.6 (7–76).	A limitation of the study is that the sample represented only a subset of ostensibly healthy women. The predominance of multiparous participants and absence of categorical analysis by age and weight constitute additional limitations. The broad range of genital measurements observed makes it challenging to delineate the boundaries of what constitutes ‘normal’ female external genitalia.
Mangla et al. (2025) [24]	Cross-sectional study (*n* = 207); outpatient obstetrics and gynaecology clinic in a hospital setting, India. Women attending the department for complaints unrelated to appearance or discomfort of external genitalia.	Premenopausal women; age range 20–48 years (mean 34.5 ± 8.1); mean BMI 24.6 ± 4.2. Parity: 0 = 18 (8.7%); 1 = 28 (13.5%); 2 = 96 (46.4%); 3 = 35 (16.9%); 4 = 30 (14.5%). Type of delivery: Both cesarean + vaginal = 13 (6.3%); Cesarean only = 17 (8.2%); Vaginal only = 81 (39.1%); None = 96 (46.4%).	Labia minora width: Mean 15.7 ± 6.8 mm (5–48 mm). Labia minora length: Mean 44 ± 09 mm (15–80 mm).	Small sample; hospital-based cohort from gynecology outpatients; limited to southern Indian women—broader data needed for racial/regional comparison. Labia minora dimensions show wide variability.
Kurtoğlu et al., (2025) [25]	Cross-sectional study (*n* = 200); outpatient gynaecology clinic in a hospital setting, Turkey. Women attending gynecological exam for reasons other than genital cosmetic surgery.	Premenopausal women; Age: Mean 33.6 ± 6.2 yrs (20–44). BMI: Mean 27.5 ± 4.8 kg/m^2^. Parity: Mean 2; 17.5% nulliparous; remainder with cesarean, vaginal, or both deliveries.	Labia minora width: Right mean 18.7 ± 9.6 mm (0–45 mm); Left mean 19.1 ± 9.4 mm (2–45 mm).	Future research comparing surgery-seeking and non-seeking women may clarify genital dimensions’ influence on self-perception (not analyzed here). Wide variation exists in genital anatomy.

(x–x) Values presented are the minimum and maximum of the range. BMI: Body Mass Index. CI: Confidence Interval.

**Table 2:** Summary of the vulvar examination methodology.

Study: Author(s), Year of Publication	Methodology Used for the Vulvar Examination
Lloyd et al. (2005) [3]	Measurements were performed under anesthesia in the lithotomy position using a disposable tape measure. Labia minora length was defined as the vertical distance from the clitoral prepuce to the posterior limit, and width as the medial distance from the base of attachment to the distal free edge at the widest point.
Basaran et al. (2008) [20]	Measurements were performed in the lithotomy position using a disposable tape measure. Labia minora length was defined as the distance from the lower border of the clitoral glans to the frenulum labiorum pudenda, and width as the medial distance from the sulcus nymphohymenalis to the free edge at its widest point.
Krissi et al., (2016) [21]	All subjects were under general anesthesia and placed in the lithotomy position. Measurements were taken using a disposable tape measure. Labia minora length was measured at the longest area, and width at the widest area. The methodology followed that of Lloyd et al. (2005) [3].
Lykkebo et al. (2017) [22]	Participants were positioned in lithotomy; measurements were obtained using a disposable tape measure. Measurements were taken without stretching the labia. Labia minora width: external aspect of the labia minora, from the infralabial sulcus to the lateral margin of the labia minora. Labia minora length: Distance from the clitoral glans to the inferior margin of the labia minora.
Kaya et al. (2020) [23]	Participants were examined in the lithotomy position. External genital measurements were taken using a digital stainless-steel Vernier caliper (precision 0.1 mm), sterilized with ethylene oxide or used with disposable glove barriers. Labia minora and majora length and width were measured bilaterally. Labia minora width was measured from the base to the widest lateral prominence, and length as the longest craniocaudal dimension.
Mangla et al. (2025) [24]	Participants were examined in the lithotomy position. External genital measurements were taken using a digital stainless-steel Vernier caliper (precision 0.1 mm). Labia minora width: medial distance from base to widest lateral prominence. Labia minora length: longest craniocaudal extension of labium minora. Bilateral measurements; mean of both sides used for analysis.
Kurtoğlu et al., (2025) [25]	Bilateral labia minora external width was measured in the lithotomy position using a disposable paper ruler, from the base at the introitus to the widest lateral prominence with minimal tension.

**Table 3:** Risk of Bias in studies.

Study	Were the Criteria for Inclusion in the Sample Clearly Defined?	Were the Study Subjects and the Setting Described in Detail?	Was the Exposure Measured in a Valid and Reliable Way?	Were Objective, Standard Criteria Used for Measurement of the Condition?	Were Confounding Factors Identified?	Were Strategies to Deal with Confounding Factors Stated?	Were the Outcomes Measured in a Valid and Reliable Way?	Was Appropriate Statistical Analysis Used?
Lloyd et al. (2005) [3]	Yes	Yes	Yes	Yes	Yes	Yes	Yes	Yes
Basaran et al. (2008) [20]	Yes	Yes	Yes	Yes	Yes	Yes	Unclear	Yes
Krissi et al. (2015) [21]	Yes	Yes	Yes	Yes	Yes	Yes	Unclear	Yes
Lykkebo et al. (2017) [22]	Yes	Yes	Yes	Yes	Yes	Yes	Yes	Yes
Kaya et al. (2020) [23]	Yes	Yes	Yes	Yes	Yes	Yes	Yes	Yes
Mangla et al. (2025) [24]	Yes	Yes	Yes	Yes	Yes	Yes	Yes	Yes
Kurtoglu et al. (2025) [25]	Yes	Yes	Yes	Yes	Yes	Yes	Yes	Yes

**Table 4:** Synthesis of the extracted data concerning the outcome measures, the length and width of the labia minora.

Study: Author(s), Year of Publication	Sample Size (*n*)	Labia Minora Length (mm). Mean	Labia Minora Length (mm). Standard Deviation	Labia Minora Width (mm). Mean	Labia Minora Width (mm). Standard Deviation
Lloyd et al. (2005) [3]	50	60.6	17.2	21.8	9.4
Basaran et al. (2008) [20]	50	55.7	11.9	17.9	4.1
Krissi et al. (2015) [21]	32	36.5	Unavailable	14.7	Unavailable
Lykkebo et al. (2017) [22]	244	42.8	Unavailable	15.5	Unavailable
Kaya et al. (2020) [23]	208	37.0	Unavailable	21.6	Unavailable
Mangla et al. (2025) [24]	207	44.0	9	15.7	6.8
Kurtoglu et al. (2025) [25]	200	Unavailable	Unavailable	18.9	Unavailable

## Data Availability

The original contributions presented in this study are included in the article. Further inquiries can be directed to the corresponding author.

## Supplementary Materials

The following supporting information can be downloaded at https://www.mdpi.com/article/10.3390/jcm15041641/s1. Table S1: PRISMA checklist.

## Abbreviations

The following abbreviations are used in this manuscript:

FGCS	Female Genital Cosmetic Surgery
ISAPS	International Society of Aesthetic Plastic Surgeons
PEO	Population (types of participants), Exposure of interest (independent variable), Outcome (dependent variable)
